# Difficulties and the Privileges of the Medical Profession

**Published:** 1852-10

**Authors:** 


*The Difficulties and the Privileges of the Medical Profession.* By HENRY CAMPBELL, M. D., Georgia.

WHEN we practiced medicine we had our full share of its difficulties—the privileges must have gone to somebody else. The address, however, is a good one, and if it can give comfort to Doctors it is a most benevolent publication.

